# Non-equivalence of Key Positively Charged Residues of the Free Fatty Acid 2 Receptor in the Recognition and Function of Agonist *Versus* Antagonist Ligands*

**DOI:** 10.1074/jbc.M115.687939

**Published:** 2015-10-29

**Authors:** Eugenia Sergeev, Anders Højgaard Hansen, Sunil K. Pandey, Amanda E. MacKenzie, Brian D. Hudson, Trond Ulven, Graeme Milligan

**Affiliations:** From the ‡ Molecular Pharmacology Group, Institute of Molecular, Cell and Systems Biology, College of Medical, Veterinary and Life Sciences, University of Glasgow, Glasgow G12 8QQ, Scotland, United Kingdom and; the § Department of Physics, Chemistry and Pharmacy, University of Southern Denmark, Campusvej 55, DK-5230 Odense M, Denmark

**Keywords:** fatty acid, G protein-coupled receptor (GPCR), homology modeling, intestinal metabolism, medicinal chemistry, 7 transmembrane domain receptor, free fatty acid

## Abstract

Short chain fatty acids (SCFAs) are produced in the gut by bacterial fermentation of poorly digested carbohydrates. A key mediator of their actions is the G protein-coupled free fatty acid 2 (FFA2) receptor, and this has been suggested as a therapeutic target for the treatment of both metabolic and inflammatory diseases. However, a lack of understanding of the molecular determinants dictating how ligands bind to this receptor has hindered development. We have developed a novel radiolabeled FFA2 antagonist to probe ligand binding to FFA2, and in combination with mutagenesis and molecular modeling studies, we define how agonist and antagonist ligands interact with the receptor. Although both agonist and antagonist ligands contain negatively charged carboxylates that interact with two key positively charged arginine residues in transmembrane domains V and VII of FFA2, there are clear differences in how these interactions occur. Specifically, although agonists require interaction with both arginine residues to bind the receptor, antagonists require an interaction with only one of the two. Moreover, different chemical series of antagonist interact preferentially with different arginine residues. A homology model capable of rationalizing these observations was developed and provides a tool that will be invaluable for identifying improved FFA2 agonists and antagonists to further define function and therapeutic opportunities of this receptor.

## Introduction

Short chain fatty acids (SCFAs)^4^ are produced in large amounts in the gut by microbial fermentation of poorly digestible carbohydrates (1–3). The predominant products are acetate (C2) and propionate (C3). SCFAs have pleiotropic effects in the body, both locally in the gut and after absorption (1–3). A broad range of these effects occurs subsequent to activation of one or both of a pair of closely related SCFA-regulated G protein-coupled receptors (GPCRs). These are designated as the free fatty acid 2 (FFA2) and free fatty acid 3 (FFA3) receptors (4–7). Mapping of key residues that contribute to the function of the SCFAs at both FFA2 and FFA3 demonstrated that mutation of either of a pair of arginine residues, one in transmembrane domain (TMD) V at position 180^5.39^ (Ballesteros and Weinstein (8) positional numbering system in superscript) and the other in TMD VII at position 255^7.35^, eliminated the agonist action of both C2 and C3 (9). Moreover, a pair of histidine residues in TMDs IV and VI, at positions 140^4.56^ and 242^6.55^, also played important roles in defining the binding pocket for the SCFAs or their function (9, 10).

There is a degree of selectivity in rank-order of potency for the SCFAs between FFA2 and FFA3 with FFA2 preferentially activated by the shorter SCFAs (4, 5) and, in general, by short carboxylic acids with *sp*^2^ or *sp*-hybridized α-carbon atoms (11). However, the selectivity is modest, and C3 is the most potent SCFA on both receptors, limiting the ability to use SCFAs to define the specific roles of FFA2 and FFA3 (12, 13). This is compounded because the absolute potency of the SCFAs also varies between human and rodent orthologs of the receptors (13). As such, the availability of higher potency and selective synthetic agonists and antagonists would greatly assist efforts to explore the specific function of FFA2 over FFA3. However, few such ligands have been described to date (14, 15). The first reported synthetic FFA2-selective agonists were a group of phenylacetamides exemplified by 4-chloro-α-(1-methylethyl)-*N*-2-thiazolylbenzeneacetamide (16–18). However, these clearly did not share the same binding site as the SCFAs as they were fully active at forms of FFA2 in which either Arg-180^5.39^ or Arg-255^7.35^ was altered to alanine (16). They also increased the observed potency of the SCFAs when co-added (16–18) and, as such, acted as both allosteric agonists and positive allosteric modulators of the SCFAs. In efforts to identify FFA2-selective agonists that share the same binding site as the SCFAs and are therefore orthosteric in action, we showed that (*R*)-3-benzyl-4-(cyclopropyl-(4-(2,5-dichlorophenyl)thiazol-2-yl)amino)-4-oxobutanoic acid (Cmp 1) is a relatively potent and highly selective agonist of both hFFA2 and murine (m)FFA2 (10). Like the SCFAs, this ligand contains a carboxylate function, and therefore, on this basis, it was not unexpected when the activity of Cmp 1 was also shown to be lacking in either the R180A^5.39^ or R255A^7.35^ mutants of hFFA2 (10).

The lack of function for Cmp 1 and the SCFAs at mutants R180A^5.39^ or R255A^7.35^ has generally been taken to suggest that the carboxylate of these ligands form ionic interactions with these arginine residues, and therefore the lack of function results from a lack of binding. However, as binding assays for FFA2 have not been available, it has not been possible to directly test this hypothesis. Therefore, in this study we have developed a radioligand binding assay for FFA2 based on a recently reported FFA2-selective antagonist, 4-[[(*R*)-1-(benzo[*b*]thiophene-3-carbonyl)-2-methyl-azetidine-2-carbonyl]-(3-chlorobenzyl)amino]-butyric acid (GLPG0974) (19), and we used this in combination with receptor mutation studies and molecular modeling to define how agonist and antagonist ligands interact with this receptor. Through this we demonstrate that although both Arg-180^5.39^ and Arg-255^7.35^ are required for agonist binding, only one of these residues is required for high affinity antagonist binding.

## Experimental Procedures

### 

#### 

##### Materials and Compounds

FFA2 ligands Cmp 1 and CATPB ((*S*)-3-(2-(3-chlorophenyl)acetamido)-4-(4-(trifluoromethyl)phenyl) butanoic acid) were synthesized as described previously (10). MeCATPB (methyl (*S*)-3-(2-(3-chlorophenyl)acetamido)-4-(4-trifluoromethylphenyl)butanoate) is an intermediate in the synthesis of CATPB (10). Racemic GLPG0974 (4-[[1-(benzo[*b*]thiophene-3-carbonyl)-2-methylazetidine-2-carbonyl]-(3-chlorobenzyl)amino]butyric acid) (19), Cmp 71 (4-(1-(benzo[*b*]thiophene-3-carbonyl)-2-methyl-*N*-(4-trifluoromethylbenzyl)azetidine-2-carboxamido)butanoic acid), MeCmp 71 (methyl 4-(1-(benzo[*b*]thiophene-3-carbonyl)-2-methyl-*N*-(4-trifluoromethylbenzyl)azetidine-2-carboxamido)butanoate), Cmp 42 (4-(1-(2-(benzo[*b*]thiophen-3-yl)acetyl)-*N*-(4-chlorobenzyl)-2-methylazetidine-2-carboxamido)butanoic acid), and MeCmp 42 (methyl 4-(1-(2-(benzo[*b*]thiophen-3-yl)acetyl)-*N*-(4-chlorobenzyl)-2-methylazetidine-2-carboxamido)butanoate) were synthesized essentially as described previously (19, 20). The identity and >95% purity of each compound was confirmed by NMR, HRMS, and HPLC.

Tissue culture reagents were from Invitrogen. Molecular biology enzymes and reagents were from Promega. The radiochemical [^35^S]GTPγS was from PerkinElmer Life Sciences. [^3^H]GLPG0974 (129 MBq/ml) was a gift of AstraZeneca (Molndal, Sweden). All other experimental reagents used were from Sigma unless indicated otherwise.

##### Plasmids and Mutagenesis

The hFFA2 or mFFA3 receptors with enhanced yellow fluorescent protein (eYFP) fused to their C termini were cloned into the pcDNA5/FRT/TO expression vector as described previously (9, 10, 13). Site-directed mutagenesis to generate the point mutations described was performed according to the QuikChange method (Stratagene, Cheshire, UK).

##### Cell Culture, Transfection, and Generation of Cell Lines

HEK293T cells were used for experiments employing transient heterologous expression. These cells were maintained in Dulbecco's modification of Eagle's medium (DMEM) supplemented with 10% fetal bovine serum, 2 mm l-glutamine, and 1× penicillin/streptomycin mixture (Sigma) at 37 °C and 5% CO_2_. Transfections were performed using polyethyleneimine, and experiments were carried out 48 h after transfection. The inducible lines are described as Flp-In^TM^ T-Rex 293 cells by Invitrogen. These cell lines were generated as described previously (10, 21) and maintained in DMEM without sodium pyruvate supplemented with 10% fetal bovine serum, 1× penicillin/streptomycin mixture, 5 μg/ml blasticidin, and 200 μg/ml hygromycin B. All experiments carried out using these cells were conducted after a 24-h treatment with 100 ng/ml doxycycline, unless otherwise stated, to induce expression of the receptor construct of interest.

##### Bioluminescence Resonance Energy Transfer (BRET) β-Arrestin-2 Recruitment Assay

HEK293T cells were co-transfected at a 4:1 ratio with plasmids encoding an eYFP-tagged form of the receptor construct of interest and a β-arrestin-2 *Renilla* luciferase (22, 23). Cells were transferred into white 96-well microtiter plates at 24 h post-transfection. At 48 h post-transfection, cells were washed, and the culture medium was replaced with Hanks' balanced salt solution immediately prior to conducting the assay. To assess the inhibitory ability of prospective antagonist ligands, test compounds were added to the cells followed by incubation for 5 min at 37 °C. To measure β-arrestin-2 recruitment to the receptor, the *Renilla* luciferase substrate coelenterazine h (Nanolight Tech, Pinetop, CA) was added to a final concentration of 2.5 μm, and cells were incubated for a further 5 min at 37 °C. Next, an EC_80_ concentration (where EC_80_ concentration is an 80% maximally effective concentration of an agonist ligand) of an appropriate agonist was added, and cells were incubated for an additional 10 min at 37 °C. BRET resulting from receptor-β-arrestin-2 interaction was assessed by measuring the ratio of luminescence at 535 and 475 nm using a PHERAstar FS plate reader fitted with the BRET1 optic module (BMG Labtech, Aylesbury, UK).

##### Intracellular Ca^2+^ Mobilization Assay

All Ca^2+^ experiments were carried out using Flp-In^TM^ T-REx^TM^ stable-inducible cell lines (24, 25). Cells were plated at 70,000/well in black 96-well plates with a clear bottom and then allowed to adhere for 3–6 h. Doxycycline was then added at 100 ng/ml concentration to induce receptor expression, and cells were maintained in culture overnight. Prior to the assay, cells were labeled for 45 min with the calcium-sensitive dye Fura-2 AM and then washed and incubated for 20 min with Hanks' balanced salt solution containing the indicated concentration of antagonist. Fura-2 fluorescent emission at 510 nm resulting from 340 or 380 nm excitation was then monitored using a Flexstation (Molecular Devices, Sunnyvale, CA) plate reader. Baseline fluorescence was measured for 16 s; test compounds were then added, and fluorescence was measured for an additional 74 s. The baseline-subtracted maximum 340/380 nm ratio obtained after the compound addition was used to plot concentration-response data.

##### [^35^S]GTPγS Incorporation Assay

Cell membranes were generated as described previously (9) from Flp-In^TM^ T-REx^TM^ cells either uninduced or treated with doxycycline (100 ng/ml unless otherwise indicated) to induce expression of the receptor construct of interest. [^35^S]GTPγS binding assays (26, 27) were performed in reactions with 5 μg of cell membrane protein pre-incubated for 15 min at 25 °C in assay buffer (50 mm Tris-HCl, pH 7.4, 10 mm MgCl_2_, 100 mm NaCl, 1 mm EDTA, 1 μm GDP, and 0.1% fatty acid-free bovine serum albumin) containing the indicated concentrations of ligands. The reaction was initiated with addition of [^35^S]GTPγS at 50 nCi per tube, and the reaction was terminated after 1 h of incubation at 25 °C by rapid filtration through GF/C glass filters using a 24-well Brandel cell harvester (Alpha Biotech, Glasgow, UK). Unbound radioligand was removed from filters by washing three times with ice-cold wash buffer (50 mm Tris-HCl, pH 7.4, and 10 mm MgCl_2_), and [^35^S]GTPγS binding was determined by liquid scintillation spectrometry.

##### cAMP Assay

All cAMP experiments were performed using Flp-In^TM^ T-REx^TM^ 293 cells able to express receptors of interest in an inducible manner. Experiments were carried out using a homogeneous time-resolved FRET-based detection kit (CisBio Bioassays, Codolet, France) according to the manufacturer's protocol. Cells were plated at 2000 cells/well in low-volume 384-well plates. The ability of agonists to inhibit 1 μm forskolin-induced cAMP production was assessed following a co-incubation for 30 min with agonist compounds, which was preceded by a 15-min pre-incubation with antagonist to allow for equilibration.

##### Extracellular-regulated Kinase 1/2 (ERK1/2) Phosphorylation Assays

Experiments were performed using a homogeneous time-resolved FRET-based detection kit (CisBio Bioassays) according to the manufacturer's protocol. Cells were plated at 15,000 cells/well in low-volume 384-well plates. After a 1-h preincubation with antagonist, agonist was added for a further 30 min, and then ERK1/2 phosphorylation was measured.

##### Procedures Applicable to All Radioligand Binding Experiments

All receptor radioligand binding experiments using [^3^H]GLPG0974 were conducted in glass tubes, in binding buffer (50 mm Tris-HCl, 100 mm NaCl, 10 mm MgCl_2_, 1 mm EDTA, pH 7.4). Membrane protein was generated from Flp-In^TM^ T-REx^TM^ cells induced to express the receptor construct of interest with 100 ng/ml doxycycline (unless otherwise stated). Nonspecific binding of the radioligand was determined in the presence of 10 μm CATPB. After the indicated incubation period at 25 °C, bound and free [^3^H]GLPG0974 were separated by rapid vacuum filtration through GF/C glass filters using a 24-well Brandel cell harvester (Alpha Biotech, Glasgow, UK), and unbound radioligand was washed from filters by three washes with ice-cold PBS. After drying (3–12 h), 3 ml of Ultima Gold^TM^ XR (PerkinElmer Life Sciences) was added to each sample vial, and radioactivity was quantified by liquid scintillation spectrometry. Aliquots of [^3^H]GLPG0974 were also quantified to define the concentration of [^3^H]GLPG0974 added per tube. In all experiments, total binding never exceeded more than 10% of that added, avoiding complications associated with free radioligand depletion (28).

##### [^3^H]GLPG0974 Association and Dissociation Kinetic Binding Assay

[^3^H]GLPG0974 dissociation and association kinetic binding assays were performed using a reverse time protocol. To assess dissociation kinetics [^3^H]GLPG0974, at approximately *K_d_* concentration, was incubated with 5 μg of membrane protein for 1 h at 25 °C in binding buffer. To induce radioligand dissociation, 10 μm CATPB was added in a time-staggered approach to capture 5–240-min time points. For determination of association kinetics, [^3^H]GLPG0974 and membrane protein were added simultaneously in a time-staggered approach to determine 5–240-min time points. All samples were then processed simultaneously.

##### [^3^H]GLPG0974 Saturation and Competition Binding Assays

To construct saturation binding isotherms, various concentrations of [^3^H]GLPG0974 were incubated with 5 μg of membrane protein potentially expressing the receptor construct of interest. For [^3^H]GLPG0974 competition binding assays, the radioligand at approximately *K_d_* concentration and varying concentrations of unlabeled ligand of choice were co-added to 5 μg of membrane protein. Incubations were performed for 2 h at 25 °C before analysis of the extent of binding of [^3^H]GLPG0974.

##### Determination of the “On” and “Off” Rates of Unlabeled Ligands through Measurement of Competition Binding Kinetics of [^3^H]GLPG0974

The kinetic binding parameters of unlabeled ligands were determined through assessment of the binding kinetics of [^3^H]GLPG0974, as described in more detail by Refs. 29 and 30. Here, binding buffer, [^3^H]GLPG0974 (at an approximate *K_d_* concentration of 10 nm), and three different concentrations of competitor (1-, 3-, and 10-fold the estimated respective *K_i_* concentration) were added simultaneously to glass tubes. At the indicated time points, membrane protein was added, and tubes were gently agitated. Three different concentrations of competitor were assessed to ensure that the rate parameters calculated were independent of ligand concentration.

##### Data Analysis and Curve Fitting

All data presented represent means ± S.E. of at least three independent experiments. All data analysis and curve fitting were carried out using the GraphPad Prism software package version 5.0b (GraphPad, San Diego). In the case of functional assays, concentration-response data were plotted on a log axis, where the untreated vehicle control condition was plotted at 1 log unit lower than the lowest test concentration of ligand and then fitted to three-parameter sigmoidal concentration-response curves. Statistical analysis of curve fit parameters was carried out by independently fitting the data from triplicate experiments and comparing the resulting curve fit values by *t* test or one- or two-way analysis of variance followed by Bonferroni, Dunnett's, or Tukey's post hoc tests as appropriate. Antagonism experiments carried out with multiple defined concentrations of antagonist were fit, where appropriate, to a global Gaddum/Schild EC_50_ shift equation to estimate p*A*_2_ values for the antagonist. In case of radioligand dissociation and association kinetic binding assays, the dissociation data were fit to a dissociation (one-phase exponential decay) model, and the association data were fit to a one-phase association model depending on the *k*_off_ value determined in parallel experiments and the measured concentration of [^3^H]GLPG0974 used. To generate saturation binding curves, the specific binding *versus* radioligand concentration was fit to a one-site specific binding model using GraphPad Prism 5, and *B*_max_ and *K_d_* values for radioligand at wild type and mutant receptors were calculated. To determine the affinity of unlabeled ligands in terms of *K_i_* values, displacement assay data were fit to an inverse three-parameter sigmoidal one-site *K_i_* value fit with the measured radioligand concentration and affinity to the respective receptor as constraints. To determine the *k*_on_ and *k*_off_ values of unlabeled ligands through competition binding kinetics of [^3^H]GLPG0974, data were fit globally using the kinetics of competitive binding equation available from GraphPad Prism, with the *k*_on_ (1.730 × 10^6^ m^−1^ min^−1^) and *k*_off_ (0.014 min^−1^) values of [^3^H]GLPG0974 entered as constraints.

##### Homology Modeling

Homology modeling of the hFFA2 receptor was performed using the hFFA1 receptor (Protein Data Bank code 4PHU) as template (31). Manual alignment was conducted using the SeaView software (32). Prior to alignment, the T4 lysozyme fusion protein construct inserted into the third intracellular loop of hFFA1 was deleted, and the final template was optimized using Protein Preparation Wizard (34), assigning bond orders and partial charges, adding hydrogen atoms, and deleting water molecules. Hydrogen bond assignment was performed at pH 7.0 using PROPKA (33). Restrained minimization until heavy atoms converged to a root mean square deviation of 0.3 Å was executed using the OPLS-2005 force field (35). The homology model of hFFA2 was constructed using Prime's homology modeling module (Prime, version 3.3, Schrödinger, LLC, New York). Mutant receptors were generated. Restrained minimization of the final structures was performed using OPLS-2005 force field in Protein Preparation Wizard (35).

##### Ligand Preparation and Induced Fit Docking

Ligands were prepared using the OPLS-2005 force field (35) in LigPrep (LigPrep, version 2.7, Schrödinger, LLC). Ionization states were generated using Epik at pH 7.0 ± 2.0; only one low-energy ring conformation per ligand was sampled (Epik, version 2.5, Schrödinger, LLC). Induced-fit docking was performed using the IFD 2006 protocol as implemented in the Schrodinger Suite version 2013-1 (Glide version 5.9, Schrödinger, LLC; Prime version 3.2, Schrödinger, LLC). Because hydrogen bonds between Arg-258^7.35^ and Asn-244^6.55^ in the hFFA1/TAK-875 co-crystal structure (31) are intact even with bound agonist, the equivalent aligned His-242^6.55^ and Arg-255^7.35^ residues were not refined during induced fit docking. Ligand conformational sampling was conducted using default settings; initial Glide docking was performed using standard settings; the maximum number of poses per ligand was restricted to 20; and trimming was allowed for Phe-87, His-140, Cys-141, Ile-145, Tyr-165, and Val-179. Re-docking was executed in Glide using standard precision mode for the five highest ranking protein-ligand complexes generated in the initial docking, which was within 30 kcal/mol of the lowest energy protein-ligand complex. Residues were refined within 5 Å of each ligand.

## Results

**FIGURE 1.**
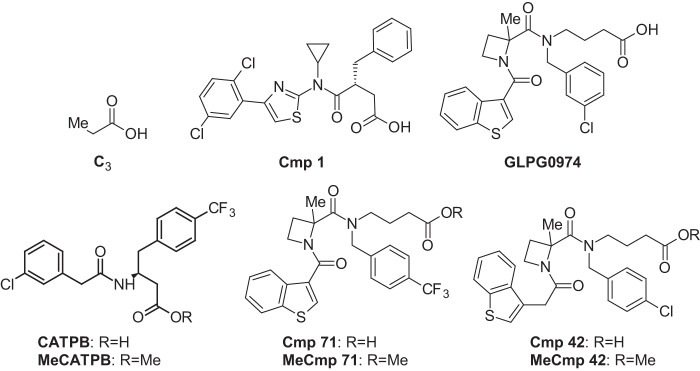
**Structures of key ligands employed.** The chemical structures of the key ligands used in the studies are shown as follows: C3; Cmp 1; GLPG0974; CATPB; MeCATPB; Cmp 42; MeCmp 42; Cmp 71; and MeCmp 71.

**FIGURE 2.**
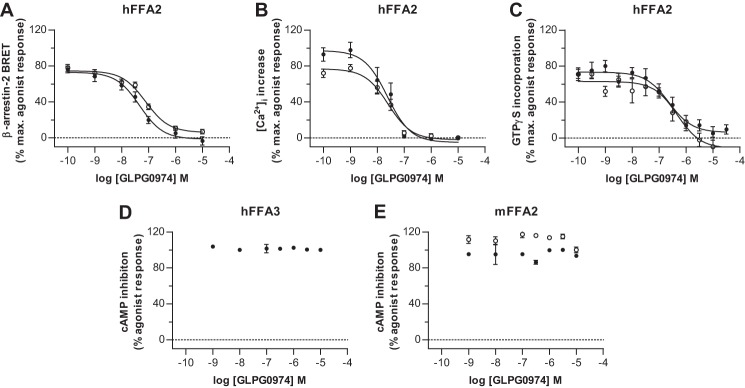
**GLPG0974 inhibits the actions of C3 and Cmp 1 at hFFA2.** hFFA2-eYFP and β-arrestin-2 *Renilla* luciferase were co-transfected transiently into HEK293T cells. The capacity of GLPG0974 to inhibit interactions between hFFA2-eYFP and β-arrestin-2 *Renilla* luciferase induced by an approximately EC_80_ concentration of C3 (3 mm) (*filled symbols*) or Cmp 1 (10 μm) (*open symbols*) was then assessed (*A*). Flp-In^TM^ T-REx^TM^ 293 cells stably harboring hFFA2-eYFP at the Flp-In^TM^ T-REx^TM^ locus were induced to express the receptor. The capacity of GLPG0974 to inhibit elevation of [Ca^2+^]*_i_* produced by EC_80_ concentrations of C3 or Cmp 1 is shown (*B*). Membranes from such induced cells were used to assess the ability of GLPG0974 to inhibit the binding of [^35^S]GTPγS stimulated by EC_80_ concentrations of C3 (300 μm) and or Cmp 1 (1 μm) (*C*). Although C3 inhibited forskolin-induced cAMP production in membranes of Flp-In^TM^ T-REx^TM^ 293 cells induced to express hFFA3-eYFP, GLPG0974 did not inhibit this (*D*). Moreover, unlike at hFFA2, GLPG0974 was unable to inhibit either C3 (*filled symbols*) or Cmp 1 (*open symbols*)-mediated inhibition of forskolin-induced cAMP production in membranes from Flp-In^TM^ T-REx^TM^ 293 cells induced to express mFFA2-eYFP (*E*).

GLPG0974 (Fig. 1) has recently been described as a selective FFA2 receptor antagonist that is able to block C2-mediated chemotaxis of human neutrophils (19). We synthesized this compound and demonstrated that it was able to antagonize, in a concentration-dependent manner, both C3 and Cmp 1-mediated activation of a form of hFFA2 that had been C-terminally tagged with enhanced yellow fluorescent protein (hFFA2-eYFP). This was the case whether employing a BRET-based β-arrestin-2 recruitment assay (Fig. 2*A*), a G_q/11_-dependent [Ca^2+^]*_i_* mobilization (Fig. 2*B*), or a G_i/o_-dependent [^35^S]GTPγS incorporation (Fig. 2*C*) assay. In each case, when employing 80% maximally effective concentrations of either C3 or Cmp 1, GLPG0974 displayed high and similar potency inhibition against each of the agonists (Table 1). GLPG0974 was highly selective for hFFA2, showing no inhibitory effect on C3-mediated activation of the closely related SCFA receptor hFFA3 (Fig. 2*D*). However, as found previously for CATPB, another FFA2 antagonist (10), GLPG0974 displayed marked species selectivity and was unable to block the effects of either C3 or Cmp 1 at mFFA2 (Fig. 2*E*).

**TABLE 1:** **GLPG0974 inhibits both C3- and Cmp 1-mediated activation of hFFA2** pIC_50_ values for GLPG0974-mediated inhibition of the effect of EC_80_ concentrations of either C3 or Cmp 1 in various functional assays were recorded. Data are mean ± S.E.

Agonist	β-Arrestin-2 BRET	[Ca^2+^]*_i_*	[^35^S]GTPγS
C3	7.43 ± 0.03	7.46 ± 0.22	6.74 ± 0.18
Cmp 1	7.15 ± 0.04	7.46 ± 0.17	6.40 ± 0.11

**FIGURE 3.**
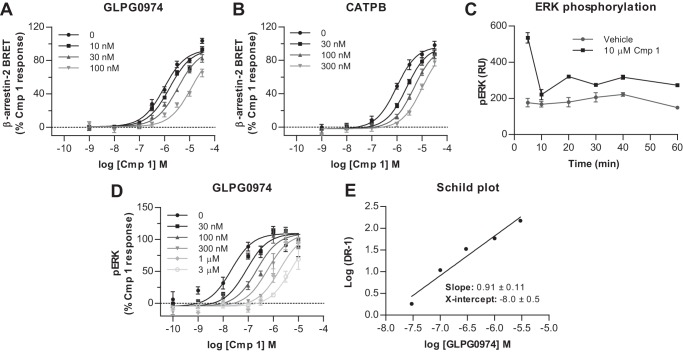
**Competitive and surmountable effects of GLPG0974 and CATPB *versus* Cmp 1.** The capacity of varying concentrations of Cmp 1 to promote interactions between hFFA2-eYFP and β-arrestin-2 *Renilla* luciferase in transiently transfected HEK293T cells and how this was altered by the co-addition of the indicated concentrations of either GLPG0974 (*A*) or CATPB (*B*) was assessed as in Fig. 2. In Flp-In^TM^ T-REx^TM^ 293 cells induced to express hFFA2-eYFP Cmp 1 (10 μm) promoted phosphorylation of the ERK1/2 MAPKs above treatment with vehicle in a manner that was sustained over time (*C*). Following pre-treatment of such cells with the indicated concentrations of GLPG0974, varying concentrations of Cmp 1 were added, and pERK1/2 levels were assessed 30 min later (*D*). Data derived from experiments akin to those in *D* are also displayed as a Schild plot (*E*). This analysis was consistent with GLPG0974 acting as a competitive antagonist of Cmp 1 and with affinity (p*A*_2_) 8.0 ± 0.5.

In the presence of increasing concentrations of GLPG0974, there was a requirement for higher concentrations of Cmp 1 to produce interactions between β-arrestin-2 and hFFA2-eYFP (Fig. 3*A*). However, higher concentrations of Cmp 1 were able to overcome the effect of GLPG0974 (Fig. 3*A*). This was also the case for antagonism of the effects of Cmp 1 by CATPB (Fig. 3*B*). Cmp 1 was also able to increase levels of phosphorylated ERK1/2 (pERK1/2) in cells expressing hFFA2 (Fig. 3*C*). Here, a rapid peak of activation, occurring within 5 min, was followed by a decline in levels of pERK1/2 to a new plateau that was maintained for at least 60 min (Fig. 3*C*). To allow effective equilibration between Cmp 1 and GLPG0974, pERK1/2 was measured 30 min after addition of the ligands. As in the β-arrestin-2 recruitment assay, increasing concentrations of GLPG0974 resulted in a requirement for higher concentrations of Cmp 1 to achieve the same level of effect (Fig. 3*D*). Both global Gaddum/Schild EC_50_ shift non-linear regression analysis (Fig. 3*D*) and presentation of the results as a Schild plot (slope 0.91 ± 0.11) (Fig. 3*E*) were consistent with GLPG0974 acting as a high affinity (p*A*_2_ estimate 7.85 ± 0.08), competitive and surmountable antagonist of hFFA2. Therefore, this antagonist is likely to bind at the orthosteric site of the receptor (10). Interestingly, despite significant structural differences, the affinity estimate for GLPG0974 was very similar to the p*A*_2_ value we obtained for CATPB (7.76 ± 0.13), the only other previously reported orthosteric antagonist of hFFA2 (Fig. 3*B*).

**FIGURE 4.**
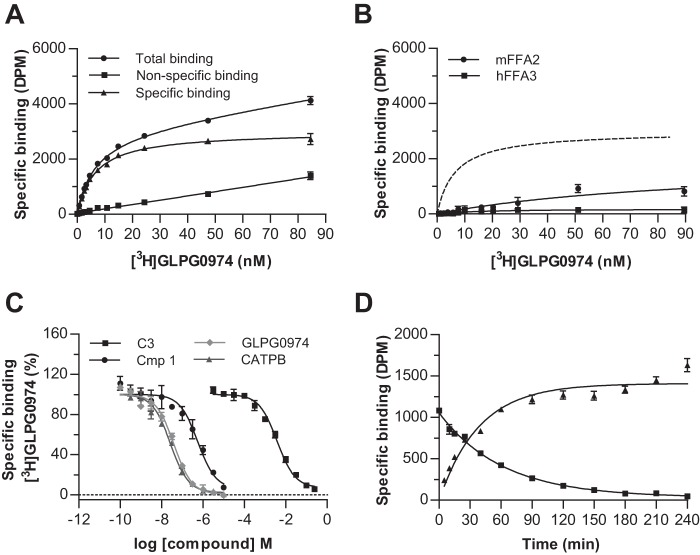
**Characteristics of [^3^H]GLPG0974 binding to wild type hFFA2.** The capacity of various concentrations of [^3^H]GLPG0974 to bind to membranes of Flp-In^TM^ T-REx^TM^ 293 cells induced to express hFFA2-eYFP is displayed (*A, circles*). Parallel experiments performed in the presence of 10 μm CATPB defined nonspecific binding of [^3^H]GLPG0974 (*A, squares*), whereas subtraction of nonspecific from total binding defined specific binding to hFFA2-eYFP (*A, diamonds*). [^3^H]GLPG0974 at a concentration up to 90 nm showed no specific binding to membranes of Flp-In^TM^ T-REx^TM^ 293 cells induced to express hFFA3-eYFP (*B*), whereas specific binding to mFFA2-eYFP was essentially linear over this concentration range and did not saturate (*B*). The capacity of varying concentrations of GLPG0974, CATPB, Cmp 1, and C3 to compete for binding of [^3^H]GLPG0974 (10 nm) is shown (*C*). Association kinetics of the specific binding of 5.75 nm [^3^H]GLPG0974 (*D*) and its subsequent dissociation (*D*) after addition of 10 μm CATPB at time 60 min allowed independent assessment of the affinity of binding of [^3^H]GLPG0974 to hFFA2 (see under “Results” for details).

To explore the mode of binding of GLPG0974 in more detail, we employed a radiolabeled form of this compound. This ligand, [^3^H]GLPG0974, displayed excellent characteristics as a radiotracer. In membranes from Flp-In^TM^ T-REx^TM^ 293 cells that had been induced to express hFFA2-eYFP [^3^H]GLPG0974 bound with high affinity (*K_d_* = 7.5 ± 0.4 nm, mean ± S.E., *n* = 4) in saturation equilibrium binding assays and with low non-specific to total binding ratios (Fig. 4*A*). Fitting of the data to both 1-site and 2-site binding models indicated no improved fit to the 2-site model, and therefore, the data are fully consistent with [^3^H]GLPG0974 binding to a single site on the receptor. As anticipated from the lack of functional effects of GLPG0974 at mFFA2 and hFFA3 (Fig. 2, *D* and *E*), [^3^H]GLPG0974 showed no specific binding to membranes of Flp-In^TM^ T-REx^TM^ 293 cells induced to express hFFA3-eYFP at concentrations up to 100 nm (Fig. 4*B*), whereas in membranes induced to express mFFA2-eYFP, a very low level of apparent specific binding was detected (Fig. 4*B*), but useful analysis of the data was not possible as the binding did not saturate, and best estimates predicted *K_d_* >150 nm. This was despite direct comparison of receptor expression, based on eYFP fluorescence, indicating that each of these constructs was expressed in amounts similar to hFFA2-eYFP.

These preliminary studies allowed [^3^H]GLPG0974 to be used in competition binding studies, which confirmed the orthosteric nature of this ligand, because both the orthosteric agonists, C3 and Cmp 1, were able to fully compete with [^3^H]GLPG0974 to bind hFFA2 (Fig. 4*C*). This was also the case for the antagonists CATPB and GLPG0974 (Fig. 4*C*). These experiments also allowed determination of the affinity for each displacing ligand. The two antagonists, CATPB (p*K_i_* = 7.87 ± 0.08) and GLPG0974 (p*K_i_* = 7.88 ± 0.08), displayed almost identical affinity (Table 2), although the affinity of Cmp 1 was ∼10-fold lower (p*K_i_* = 6.91 ± 0.12). Furthermore, as anticipated from previous functional studies (9–11), C3 displayed very modest affinity (p*K_i_* = 2.96 ± 0.11) at hFFA2. [^3^H]GLPG0974 also proved useful in assessing the binding kinetics of the ligand (Fig. 4*D*), indicating that its rate of association was 1,730,000 ± 74,000 m^−1^ min^−1^ (Fig. 4*D*), and its rate of dissociation was 0.014 ± 0.001 min^−1^ (Fig. 4*D*). Using these values to independently determine the affinity of [^3^H]GLPG0974 yielded a *K_d_* of 8.1 ± 0.9 nm (mean ± S.E., *n* = 3), a value similar to that obtained from the saturation binding studies (Fig. 4*A*).

**TABLE 2:** **Estimated affinity of ligands at wild type and orthosteric binding pocket mutants of FFA2, competition binding studies using [^3^H]GLPG0974** The ability of CATPB and GLPG0974 to compete with [^3^H]GLPG0974 to bind to wild type hFFA2 and each of the indicated mutants of hFFA2 was assessed. Data are presented as calculated affinity constant (p*K_i_*) values (mean ± S.E.). ***, *p* < 0.001. One-way analysis of variance was followed by Dunnett's test with WT as reference.

Receptor	CATPB	GLPG0974
WT	7.87 ± 0.08	7.88 ± 0.08
R255A^7.35^	6.98 ± 0.06***	7.59 ± 0.09
R180A^5.39^	7.32 ± 0.06***	7.14 ± 0.06***
H242A^6.55^	7.63 ± 0.07	8.04 ± 0.04
H140A^4.56^	7.99 ± 0.09	8.56 ± 0.10***

**FIGURE 5.**
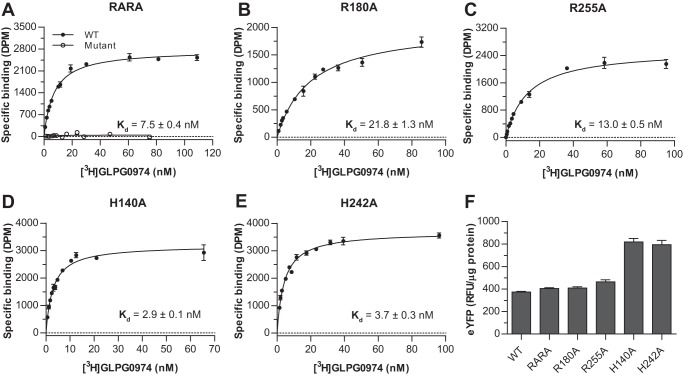
**Binding characteristics of [^3^H]GLPG0974 to orthosteric binding site mutants of hFFA2.** Specific binding of [^3^H]GLPG0974 was assessed as in Fig. 4 in membranes from Flp-In^TM^ T-REx^TM^ 293 cells induced to express wild type or R180A^5.39^/R255A^7.35^ (*RARA*) (*A*), R180A^5.39^ (*B*), R255A^7.35^ (*C*), H140A^4.56^ (*D*), or H242A^6.55^ (*E*) hFFA2-eYFP. Inserted values are ligand *K_d_* ± S.E. No specific binding to R180A^5.39^ R255A^7.35^ hFFA2-eYFP could be measured. Membranes from cells expressing each of the forms above were assessed for relative levels of expression based on fluorescence corresponding to eYFP (*F*). *RFU,* relative fluorescence units.

The FFA2 agonists C3 and Cmp 1 both contain a carboxylate that is believed to be coordinated by two arginine residues, Arg-180^5.39^ and Arg-255^7.35^, in the receptor. This is based on the observation that both agonists lack function at individual alanine mutants of either of these arginines (9, 10) and that replacement of the carboxylate of Cmp 1 with either methyl ester or *tert*-butyl ester groups also eliminated function (10). Although no atomic level structure of FFA2 is currently available, in previously generated homology models (9), these residues are closely apposed (see below). Because we have found both CATPB and GLPG0974 to also be orthosteric, and both contain a carboxylate, a reasonable hypothesis was that this moiety would bind in a similar manner for these antagonists as it does for the agonists. Consistent with this hypothesis, high affinity, specific binding of [^3^H]GLPG0974 was lacking in membranes from cells induced to express a double R180A^5.39^/R255A^7.35^ mutant of hFFA2 (Fig. 5*A*). However, unexpectedly, the single R180A^5.39^ and R255A^7.35^ mutant forms of hFFA2 each retained binding of [^3^H]GLPG0974 with only modest, although statistically significant (*p* < 0.001), reductions in affinity (Fig. 5, *B* and *C*). Of note, the reduction in affinity was more pronounced for the R180A^5.39^ mutant compared with the R255A^7.35^ variant (3-fold *versus* 1.7-fold), perhaps suggesting a somewhat more important role for Arg-180^5.39^ in the binding of this ligand. We also assessed the ability of [^3^H]GLPG0974 to bind to either H140A^4.56^ (Fig. 5*D*) or H242A^6.55^ (Fig. 5*E*) hFFA2 mutants, as these residues are also known to play important roles in FFA2 agonist binding and/or function (9, 10). [^3^H]GLPG0974 bound effectively to both of these mutants and, in each case, with slightly higher affinity than to the wild type hFFA2 construct (Fig. 5, *D* and *E*). To confirm that the lack of specific binding of [^3^H]GLPG0974 to the dual R180A^5.39^/R255A^7.35^ mutant of hFFA2-eYFP did not simply reflect lack of expression, levels of eYFP fluorescence from membranes expressing wild type and each hFFA2-eYFP mutant construct were measured and compared (Fig. 5*F*). This confirmed that all mutants were expressed and that specifically the R180A^5.39^/R255A^7.35^ double mutant was expressed at levels comparable with the wild type construct to which specific binding of [^3^H]GLPG0974 could be measured effectively.

**FIGURE 6.**
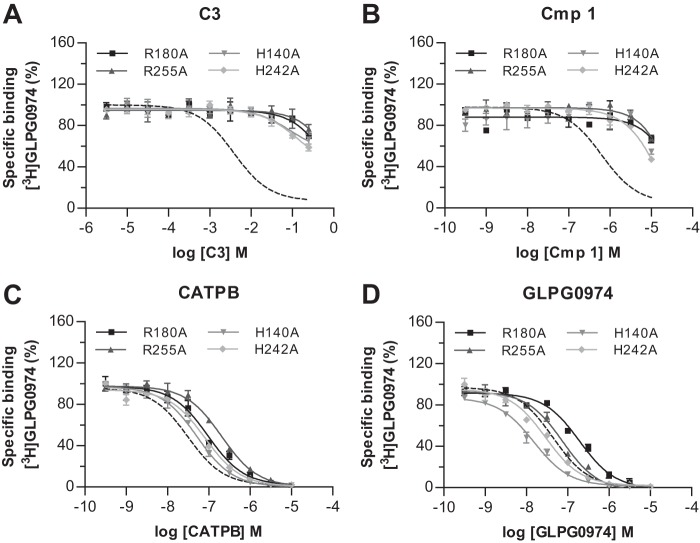
**Agonist but not antagonists of hFFA2 show markedly reduced ability to complete with [^3^H]GLPG0974 at receptor binding site mutants.** The capacity of C3 (*A*), Cmp 1 (*B*), CATPB (*C*), and GLPG0974 (*D*) to compete with [^3^H]GLPG0974 for binding to R180A^5.39^ (*squares*), R255A^7.35^ (*triangles*), H140A^4.56^ (*inverted triangles*), or H242A^6.55^ (*diamonds*) hFFA2-eYFP is shown. The effect of each ligand at wild type hFFA2-eYFP is illustrated by the *broken lines*.

Because [^3^H]GLPG0974 retained affinity for each of the orthosteric binding site single mutants, this compound allowed us to examine the importance of each of these residues in the binding of other ligands to hFFA2. Initially, we explored the ability of the agonists C3 (Fig. 6*A*) and Cmp 1 (Fig. 6*B*) to compete with [^3^H]GLPG0974 to bind to each individual mutant. These experiments demonstrated that each of the mutations markedly reduced the affinity of both C3 and Cmp 1 as only minimal competition could be observed even at the highest agonist concentrations that could be employed (Fig. 6, *A* and *B*). Such findings support the conclusion that the loss of function of these agonists previously described at each of these mutations (9, 10) results from a substantial reduction in binding affinity and is not simply a loss of ability to activate the receptor.

In contrast, when we examined the ability of the antagonist CATPB to compete with [^3^H]GLPG0974 at the single FFA2 mutants, much more modest effects were observed (Fig. 6*C* and Table 2). Indeed, no reduction in CATPB affinity was observed for H140A^4.56^ and H242A^6.55^. Moreover, although both the R180A^5.39^ (*p* < 0.001) and the R255A^7.35^ (*p* < 0.001) mutations did produce statistically significant reduction in affinity, these were still modest (7.8-fold at R255A^7.35^ and 3.5-fold at R180A^5.39^) compared with the loss of affinity of the two agonists at these mutants. Similar experiments were conducted using GLPG0974 as competitor (Fig. 6*D* and Table 2). No significant loss of affinity for GLPG0974 was observed at either R255A^7.35^ or H242A^6.55^, whereas a significant (*p* < 0.001) increase in affinity was observed for H140A^4.56^. Notably, the affinity of GLPG0974 was significantly reduced at R180A^5.39^ hFFA2 (*p* < 0.001), in this case by 5.5-fold (Fig. 6*D* and Table 2), suggesting this residue is likely the most significant arginine for GLPG0974 binding. These results, importantly, are consistent with the saturation binding studies that also implicated Arg-180^5.39^ as the more important of the two key arginine residues for [^3^H]GLPG0974 binding. Taken together, analysis of the affinity of GLPG0974 and CATPB at these mutants suggests that, despite having similar overall affinity, there is a difference in which of the two key arginine residues is most important for binding, Arg-180^5.39^ for GLPG0974 *versus* Arg-255^7.35^ for CATPB.

**FIGURE 7.**
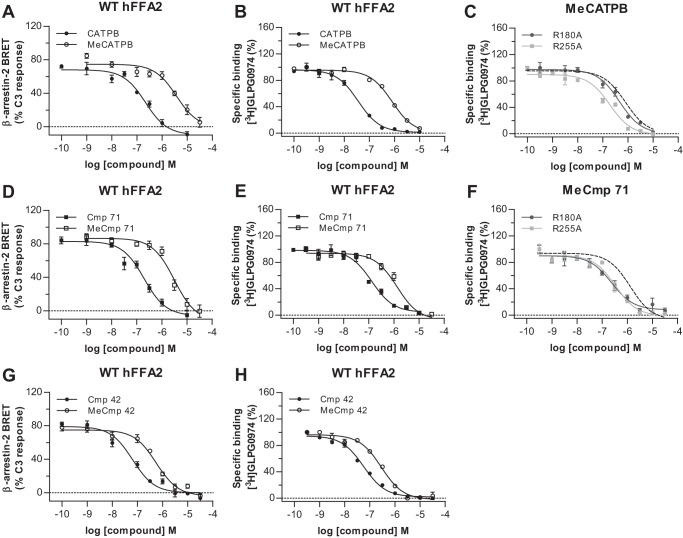
**Methyl esters of hFFA2 antagonists display lower functional potency and affinity than the corresponding carboxylates at hFFA2.** The ability of various concentrations of CATPB (*filled symbols*)/MeCATPB (*open symbols*) (*A* and *B*), of Cmp 71 (*filled symbols*)/MeCmp 71 (*open symbols*) (*D* and *E*), of Cmp 42 (*filled symbols*)/MeCmp 42 (*open symbols*) (*G* and *H*) to inhibit C3-mediated interactions between hFFA2-eYFP and β-arrestin-2-*Renilla* luciferase (*A, D,* and *G*) or compete with [^3^H]GLPG0974 for binding to wild type hFFA2-eYFP (*B, E,* and *H*) was assessed. The effects of R180A^5.39^ or R255A^7.35^ mutation on the ability of MeCATPB (*C*) or MeCmp 71 (*F*) to compete with [^3^H]GLPG0974 to bind is also shown. The effect of each ligand at wild type hFFA2-eYFP is illustrated by the *broken lines*.

As we were surprised by the relatively minor effect of removing the positively charged arginine residues (at least individually) in hFFA2 on binding of the two antagonists, we next addressed whether this reflected limited importance of the carboxylate functional group for binding of these antagonists. We considered this primarily because in previous models of FFA2 agonist binding, the carboxylate functionality has generally been predicted to form an ionic interaction with the key arginine residues of the receptor (9, 10). Therefore, we synthesized a variant of CATPB in which the carboxylic acid group was replaced with a methyl ester (MeCATPB) (Fig. 1). At wild type hFFA2 MeCATPB also functioned as an antagonist, able to block the ability of C3 to promote recruitment of β-arrestin 2 in a concentration-dependent manner (Fig. 7*A*). However, the potency of MeCATPB to do so was significantly less than CATPB (Fig. 7*A*). MeCATPB was also able to compete with [^3^H]GLPG0974 for binding, but it did so with a 13-fold reduction in affinity (*p* < 0.01) compared with the carboxylate-containing antagonist CATPB (Fig. 7*B* and Table 3). This reduction in affinity appeared to result from a loss of ionic interaction with the key arginine residues because, unlike for CATPB, there was no significant decrease in affinity of MeCATPB at either the R180A^5.39^ or R255A^7.35^ mutants compared with the wild type receptor (Fig. 7*C* and Table 3). Indeed, at R255A^7.35^ MeCATPB displayed a modest trend, that was, however, not statistically significant, toward an increase in affinity compared with the wild type receptor (Fig. 7*C* and Table 3). This may reflect that the substitution of Arg to Ala opens up the binding pocket to allow more rapid access of this ligand (see below). Next, to extend these analyses and to examine the importance of the carboxylate to antagonists structurally related to GLPG0974, we synthesized a carboxylate (Cmp 71)/methyl ester (MeCmp 71) ligand pair (Fig. 1) based on a representative compound in the GLPG0974 chemical series. These two compounds both contain a *p*-trifluoromethylbenzyl moiety instead of the *m*-chlorobenzyl group of GLPG0974 (Fig. 1). MeCmp 71 also acted as an antagonist of C3 (Fig. 7*D*), and as with the MeCATPB/CATPB pair, MeCmp 71 was significantly less potent than Cmp 71 (Fig. 7*D*). As anticipated from the above data, MeCmp 71 was also able to compete with [^3^H]GLPG0974 for binding to hFFA2, but it did so with significantly (*p* < 0.001) reduced affinity (17-fold) compared with Cmp 71 (Fig. 7*E* and Table 3). As with MeCATPB, there was no reduction of the binding affinity of MeCmp 71 to either the R180A^5.39^ or R255A^7.35^ hFFA2 single mutations compared with wild type hFFA2, and indeed, there was a significant increase in affinity (*p* < 0.01) at both (Fig. 7*F* and Table 3). This further supports the hypothesis that it is a loss of an ionic interaction with one or the other of these arginine residues that accounts for the reduced affinity of the methyl ester derivatives. As a further test of the contribution of the carboxylate moiety to binding affinity and function in this chemical series, we synthesized a further carboxylate (Cmp 42)/methyl ester (MeCmp 42) pair from this chemical series (Fig. 1) and performed both functional (Fig. 7*G*) and binding studies (Fig. 7*H*). Once again, in both situations the methyl ester was less effective than the carboxylate.

**TABLE 3:** **Estimated affinity of carboxylate and methyl ester pairs of FFA2 antagonists at wild type, R180A^5.39^, and R255A^7.35^ hFFA2** The ability of CATPB, MeCATPB, Cmp 71, and MeCmp 71 to compete with [^3^H]GLPG0974 to bind to wild type (WT), R180A^5.39^, or R255A^7.35^ hFFA2 is shown. Data are calculated affinity constant (p*K_i_*) values (mean ± S.E.). One-way analysis of variance was followed by Dunnett's test *versus* WT (*, *p* < 0.05; **, *p* < 0.01; *p* < ***, *p* < 0.001).

Receptor	CATPB	MeCATPB	Cmp 71	MeCmp 71
WT	7.87 ± 0.08	6.74 ± 0.14*^a^*	7.39 ± 0.04	6.22 ± 0.09*^a^*
R255A^7.35^	6.98 ± 0.06***	7.08 ± 0.10	7.06 ± 0.09*	6.80 ± 0.08**
R180A^5.39^	7.32 ± 0.06**	6.52 ± 0.14	7.01 ± 0.10*	6.89 ± 0.07**

*^a^ p* < 0.001 for methyl ester *versus* carboxylate-containing antagonist at wild type. Two-way analysis of variance with subsequent Bonferroni was used to compare CATPB/Cmp 71.

**FIGURE 8.**
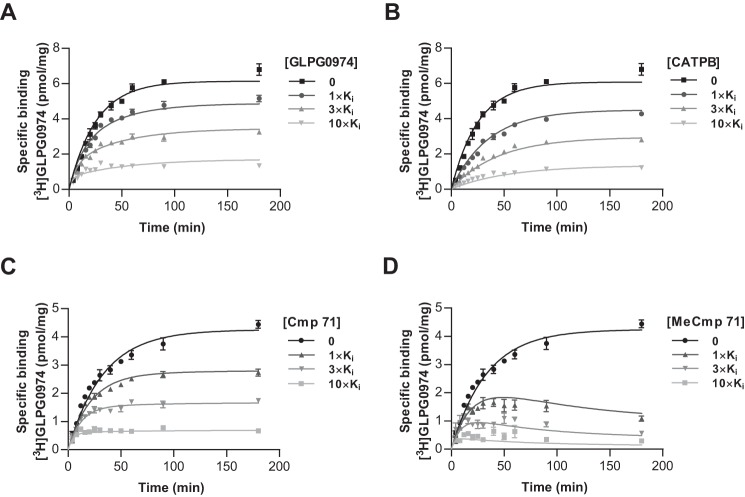
**Effects on the binding kinetic of [^3^H]GLPG0974 demonstrate markedly different on and off rates for the antagonist series.** The specific binding of [^3^H]GLPG0974 (10 nm) to membranes of Flp-In^TM^ T-REx^TM^ 293 cells induced to expressed hFFA2-eYFP was assessed at a range of time points. The studies were performed in the absence or presence of the indicated concentrations of either GLPG0974 (*A*) or CATPB (*B*). Visual analysis shows clearly that the binding of [^3^H]GLPG0974 was slower in the presence of CATPB than in the presence of GLPG0974, reflecting the faster on rate kinetic of CATPB. Analysis as shown previously (28, 29) allowed estimation of both on and off rates for the two antagonists (see under “Results” and Table 4 for details). *C* and *D,* equivalent studies were performed with Cmp 71 (*C*) and MeCmp 71 (*D*) and illustrate the markedly slower on rate kinetic of the methyl ester of the pair (Table 4).

Having established that an ionic interaction with either Arg-180^5.39^ or Arg-255^7.35^ contributes significantly to the binding affinity of the hFFA2 antagonists, we next wished to establish how these interactions affected binding affinity, *i.e.* whether they were primarily altering binding on-rate or off-rate of these ligands. For this, we used [^3^H]GLPG0974 to first establish binding kinetics for unlabeled GLPG0974 (Fig. 8*A*) and CATPB (Fig. 8*B*) at wild type hFFA2. These experiments demonstrated that CATPB had both on- and off-rates that were greater than GLPG0974, despite each compound having similar overall affinity for wild type hFFA2 (Table 4), further demonstrating differences in the mode of binding of these two antagonists to hFFA2. To establish the contribution of the ionic interaction to binding kinetics, we next compared the on- and off-rates of Cmp 71 (Fig. 8*C*) with MeCmp 71 (Fig. 8*D*). Notably, the reduction in affinity for MeCmp 71 was the result of a greater than 10-fold decrease of the on-rate of this compound with little change in off-rate (Table 4), suggesting that the electrostatic interaction primarily contributes to recruit the compound into the binding pocket. By contrast, the lower affinity of [^3^H]GLPG0974 to bind to both R180A^5.39^ and R255A^7.35^ hFFA2 largely reflected increases in the ligand off-rate from these variants. This was particularly pronounced for the R180A^5.39^ mutant that displayed more than 15-fold increase (Table 5), suggesting that the interaction of the ligand carboxylate with this binding pocket residue acts to hold the ligand once initial engagement has taken place.

**TABLE 4:** **Kinetic analysis of the binding of antagonist ligands to wild type hFFA2** Ligand on and off rates were calculated from kinetic binding assays as illustrated in Fig. 8. Data are means ± S.E. *n* > 4. *K_d_* values (*k*_off_/*k*_on_) were assessed from these values.

	GLPG0974	CATPB	Cmp 71	MeCmp 71
*k*_on_	1,220,000 ± 87,000	6,360,000 ± 1,540,000	398,000 ± 16,200	26,900 ± 9800
*k*_off_	0.021 ± 0.002	0.094 ± 0.026	0.016 ± 0.001	0.011 ± 0.007
*K_d_*	17.2 ± 0.6 nm	14.5 ± 0.7 nm	39.9 ± 1.7 nm	638 ± 259 nm

**TABLE 5:** **Kinetic analysis of the binding of [^3^H]GLPG0974 to orthosteric binding site mutants of hFFA2** One-way analysis of variance was followed by Dunnett's test with WT as reference (***, < *p* < 0.001).

	WT	R180A^5.39^	R255A^7.35^
*k*_on_	1,730,000 ± 74,000	6,794,000 ± 3,388,000	3,480,000 ± 167,000
*k*_off_	0.014 ± 0.001	0.221 ± 0.004***	0.107 ± 0.009***
*K_d_*	8.1 ± 0.9 nm	32.5 ± 16.8 nm	30.7 ± 4.1 nm

**FIGURE 9.**
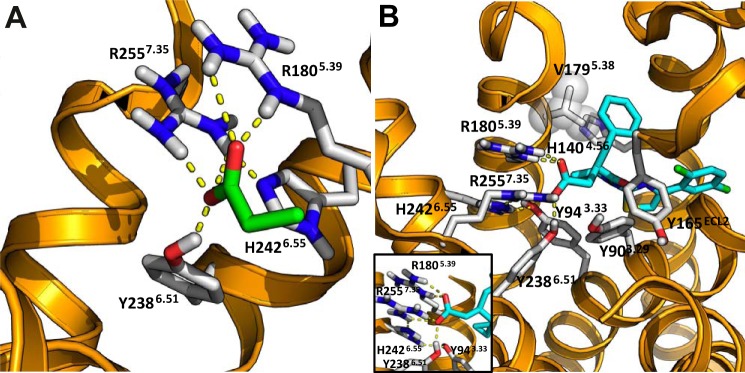
**Interaction of key orthosteric binding pocket residues with FFA2 agonists.** Docking of C3 (*A*) or Cmp 1 (*B*) into a homology model of wild type hFFA2 is illustrated. Representative binding poses show interactions with both Arg-180^5.39^ and Arg-255^7.35^, both of which residues are necessary for these agonists to bind and activate hFFA2. The models also highlight the contribution of His-242^6.55^ for organization of the binding pocket for the carboxylate of each agonist by interacting with Arg-255^7.35^. The docked binding mode of Cmp 1 is further supported by important amino acids (including Tyr-90^3.33^, His-140^4.56^, Tyr-165^ECL2^, Val-179^5.38^, and Tyr-238^6.51^), which in functional assays have been shown to affect the ability of Cmp 1 to activate hFFA2 (10). The *inset* to *B* shows greater detail of ionic interactions. In particular, the Arg-255–His-242 dyad and the Arg-255–His-242–Tyr-94 triad are highlighted.

Finally, having observed key differences in binding between agonists (require each of the Arg and His residues) and antagonists (require only a single Arg residue) and, indeed, between the two different series of antagonists, which demonstrated a preference for different Arg residues, we attempted to assess whether molecular modeling studies could rationalize these experimental observations. Models of hFFA2 were generated based on the available atomic level structure of the closely related receptor hFFA1 complexed to the allosteric partial agonist ligand TAK-875 (Protein Data Bank code 4PHU) (31). Initially, docking studies were carried out to explore possible binding modes for the agonists C3 (Fig. 9*A*) and Cmp 1 (Fig. 9*B*). Consistent with the experimental data indicating that agonists require both Arg-180^5.39^ and Arg-255^7.35^ to bind, representative poses for both C3 and Cmp 1 enabled the agonists to form electrostatic interactions with both of the arginine residues simultaneously and to engage in hydrogen bonding interactions with the conserved Tyr238^6.51^ (Fig. 9, *A* and *B*). In addition, this model suggested that an interaction formed between His-242^6.55^ and Arg-255^7.35^ that was important in positioning Arg-255^7.35^ within the binding pocket (Fig. 9, *A* and *B*). This is consistent with the finding that C3 and Cmp 1 display markedly lower affinity for the H242A^6.55^ mutant (Fig. 6, *A* and *B*). Moreover, His-140^4.56^ and Val-179^5.38^, both of which previously have been reported to significantly reduce the potency of Cmp 1 in functional assays (10), were found to be in proximity to the phenyl group of Cmp 1 in the proposed binding pose (Fig. 9*B*). Further validation for this model came from the observation that it also predicted close proximity of Cmp 1 to several other residues (Fig. 9*B*), including Tyr-90^3.33^, Tyr-165^ECL2^, and Tyr-238^6.51^, that, when mutated, alter the potency of the ligand in functional assays (10).

**FIGURE 10.**
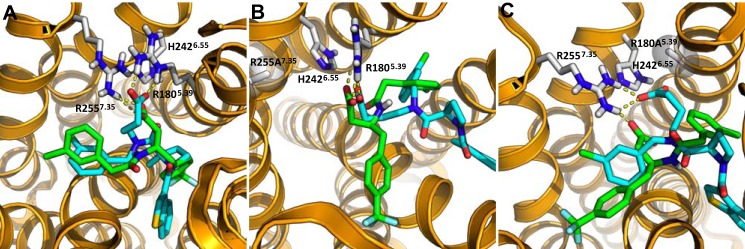
**Selective interactions of CATPB and GLPG0974 with Arg-180^5. 39^ and Arg-255^7.35^.** Representative poses of CATPB (*green*) and GLPG0974 (*cyan*) in hFFA2 (*A*) enabled ionic interactions between GLPG0974 and Arg-180^5.39^ and between CATPB and Arg-255^7.35^. The two antagonists, originating from distinct chemical series, attain similar poses despite their structural differences. In the point mutant R255A^7.35^ hFFA2 model, CATPB and GLPG0974 were able to retain similar docked binding poses as the ones observed in wild type hFFA2 while interacting electrostatically with Arg-180^5.39^ (*B*). In the point mutant R180A^5.39^, both antagonists are now able to engage Arg-255^7.35^ (*C*).

Docking studies were then carried out for CATPB and GLPG0974. These studies resulted in closely overlapping poses for these two antagonists, despite their structural differences (Fig. 10*A*). Interestingly, each compound appeared to form an electrostatic interaction with mainly one of the two key Arg residues. Specifically, GLPG0974 appeared to favor interaction with Arg-180^5.39^, whereas CATPB favored interaction with Arg-255^7.35^, observations seemingly supported by the relative effects of mutation of each of these residues on the affinity of the ligands in the binding studies (Table 2). Additional support for the model was also provided when considering results with the H242A^6.55^ mutant as, although the results did not reach statistical significance, this mutation tended to reduce affinity for CATPB but not GLPG0974 (Table 2). This observation is entirely consistent with the notion that interactions between His-242^6.55^ and Arg-255^7.35^ act to position and orient Arg-255^7.35^ as the more important arginine residue for recognition of CATPB.

**FIGURE 11.**
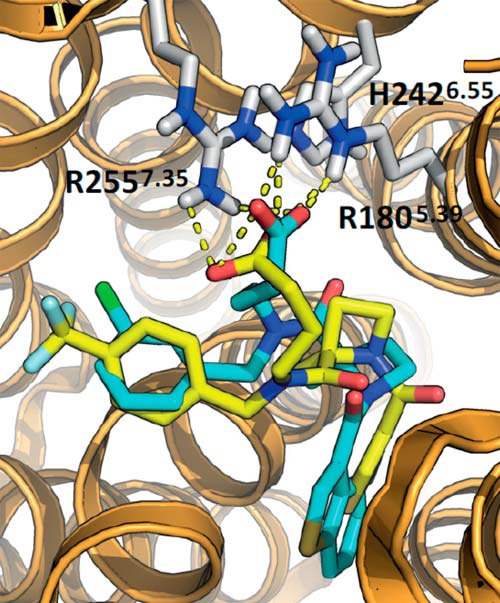
**Comparison of docked poses of Cmp 71 and GLPG0974 in hFFA2.** The two antagonists Cmp 71 (*yellow*) and GLPG0974 (*cyan*), originating from the same chemical series, attain similar docked poses within the binding site of hFFA2. Representative poses of Cmp 71 and GLPG0974 in hFFA2 enabled ionic interactions between GLPG0974 and Arg-180^5.39^ and between Cmp 71 and both Arg-180^5.39^ and Arg-255^7.35^.

These modeling results suggested that when only one of the key arginine residues is mutated, a substantial loss of binding affinity is not observed for the hFFA2 antagonists because interaction with the second, less favorable, arginine may form to compensate. To explore this hypothesis, we incorporated the R255A^7.35^ mutation into the model and examined binding poses of each antagonist (Fig. 10*B*). In doing so, we found that both CATPB and GLPG0974 were able to retain similar orientations within this mutant receptor as compared with their binding pose in the wild type, each by forming an interaction with the Arg-180^5.39^. Equivalent modeling of the R180A^5.39^ mutant (Fig. 10*C*) also indicated a capacity of the carboxylate of both antagonists to now interact in a similar manner with Arg-255^7.35^. This confirms, at least in the model, that compensatory interactions may occur with the remaining arginine if the other is eliminated. Because Cmp 71 contains a *p*-trifluoromethylbenzyl group in place of the *m*-chlorobenzyl found in GLPG0974, we also modeled potential docking poses of this ligand as a comparison (Fig. 11). Representative poses of Cmp 71 in hFFA2 were similar to those for GLPG0974 but enabled ionic interactions with both Arg-180^5.39^ and Arg-255^7.35^.

## Discussion

A thorough understanding of the basis of interactions between small molecule ligands and their target proteins is central to efforts in chemical biology. Moreover, in the case of proteins that may be targets for the design of small molecule ligands to treat disease, a detailed level of understanding is integral for designing selectivity over interactions with closely related proteins and, potentially, to limit toxicity or other side effects (36). GPCRs have been the most successfully targeted group of proteins for therapeutic use. This focus means that for family members that have attracted a great level of interest for many years, such as receptors for adrenaline or for acetylcholine, a vast body of information is available on modes of ligand binding. Indeed, in these cases atomic level structures of receptors with bound agonist and/or antagonist ligands have largely confirmed predictions developed over many years of studies employing, for example, receptor mutagenesis (37–40). Moreover, the availability of a large number of ligands, often with substantial compound series containing detailed structure-activity information, has greatly facilitated understanding. However, even for relatively well characterized GPCRs, surprises in the mode of binding of certain ligands have been produced when atomic level structures have become available (31, 40). There remain a large number of GPCRs for which no atomic level structural information is available and where even ligand pharmacology is extremely limited. In such cases, analysis of the details of the ligand binding pocket(s) and the designed development of improved ligands is exceptionally challenging.

In recent years, it has become clear that a number of GPCRs are activated by ligands that are also metabolic intermediates (41–44). The group of GPCRs that respond to SCFAs, including C2 and C3, have been suggested to be potential therapeutic targets in relation to both metabolic diseases (42–44) and, particularly, inflammatory conditions (1, 45, 46). Indeed, observations of the blockade of neutrophil chemotaxis by the FFA2 receptor antagonist GLPG0974 was taken as a useful, and potentially prognostic, indicator of the potential of this compound to improve lower gut inflammatory disorders, including ulcerative colitis (19). This resulted in GLPG0974 being used in “first in man” clinical trials of an FFA2 antagonist (19). As also noted by Ref. 19, we confirmed that GLPG0974 had no ability to block agonist effects at the other SCFA GPCR, FFA3. Moreover, as we have also shown for CATPB (10), GLPG0974 displayed substantial species selectivity and was unable to inhibit agonist activation of mFFA2. As these species orthologs show modest variation in regions close to the binding pocket for SCFAs then, in the future, the availability of [^3^H]GLPG0974 should allow the basis for these difference(s) to be probed via generation of a variety of point mutations and species ortholog chimeras.

SCFAs, but not the corresponding amides, are agonists at FFA2 (9). As such, the importance of the carboxylate in either or both binding to or activation of the receptor is evident. Indeed, that formic acid (C1) has some level of potency at FFA2 demonstrates that the carboxylic acid is the only necessary requisite for activation of the receptor (11, 47, 48). A reliance exclusively on functional end points cannot, however, separate these possibilities. Initial analysis of the mode of binding of the SCFAs focused on positively charged amino acids shared between FFA2 and FFA3 that might interact with the carboxylate of the ligands (9). Mutation to alanine of either Arg-180^5.39^ or Arg-255^7.35^ in hFFA2 eliminated responses to the SCFAs (9) leading to the conclusion that both were of equal importance. Furthermore, this view was extended by the first report of a synthetic orthosteric agonist of FFA2, Cmp 1, which also contained a carboxylate moiety and also lacked function at both R180A^5.39^ hFFA2 and R255A^7.35^ hFFA2 (10).

Key to the current reassessment of these conclusions was synthesis of the hFFA2 radiotracer [^3^H]GLPG0974. Although unable to bind with high affinity to the double mutant R180A^5.39^/R255A^7.35^ hFFA2, this ligand did bind to both the R180A^5.39^ and R255A^7.35^ hFFA2 single mutants with only slightly reduced affinity compared with the wild type receptor. This allowed the contribution of each of these positively charged residues to be evaluated separately. Although rather modest reduction in affinity was noted for both CATPB and GLPG0974 at these mutants, binding of agonist ligands, including Cmp 1, which is relatively potent and displays affinity close to 100 nm at wild type FFA2, was all but lost following mutation of either Arg-180^5.39^ or Arg-255^7.35^. This indicates that both of these residues are integral to recognition of agonist ligands. This is supported by the fact that the endogenous agonists of this receptor are SCFAs and that the only currently reported orthosteric agonists contain a carboxylate moiety. Revealingly, mutation of His-242^6.55^ to Ala all but ablated agonist affinity at hFFA2. Based on the atomic level structure of hFFA1 bound by the agonist TAK-875 (31), a key role of the equivalent Asn-244^6.55^ residue in FFA1 is to provide stabilization of the arginine residue at position 7.35. Assuming this is also true in hFFA2, then destabilization of the position of this Arg (sequence position 255) would be consistent with the observed loss of interaction and function of orthosteric agonists. Indeed, this arrangement was observed between Arg-255^7.35^ and His-242^5.39^ after induced fit docking without manual interference or refinement of the residues.

Antagonists are defined by their capacity to bind to but lack the ability to activate their target receptor. Although both GLPG0974 and the previously described hFFA2 antagonist CATPB contain a carboxylate, it had been unclear whether this interacted with either or both Arg-180^5.39^/Arg-255^7.35^ and whether this was integral to provide affinity. However, as [^3^H]GLPG0974 could bind to both R180A^5.39^ and R255A^7.35^ hFFA2 variants with only rather modestly reduced affinity compared with wild type, this seemed unlikely. To assess this more directly and to define the contribution of the carboxylate to binding affinity, we generated carboxylate and methyl ester pairs of exemplars of the two described classes of FFA2 antagonists. Although there was a clear reduction in affinity of each methyl ester at wild type FFA2 compared with the corresponding carboxylate-containing ligand, this effect was in the region of 10–15-fold, and no reduction in affinity for the methyl esters *versus* the carboxylate was observed at R255A^7.35^ FFA2. Indeed, the affinity of the methyl esters was slightly higher at this mutant. This may possibly reflect opening up extra space in the binding pocket by removing the size as well as the charge of the arginine residue.

Although initial homology models that were used to predict the mode of binding of the SCFAs to FFA2 indicated a contribution of both Arg-180^5.39^ and Arg-255^7.35^ (9), the very small size of the ligands and low potency limited significantly further consideration. As such, even though the ligands remained small and displayed poor potency, the identification of a series of small carboxylic acids that activate FFA2 with significant selectivity over FFA3 (11), coupled with further mutagenesis studies (11), resulted in a clearer understanding of the orthosteric binding pocket of the receptor. This was further enhanced by synthesis and testing of the first described selective and moderately potent orthosteric agonists of hFFA2, including Cmp 1 used herein (10). Key interactions of the carboxylate of this ligand with both Arg-180^5.39^ and Arg-255^7.35^ were both predicted and verified as Cmp 1 lacked function after mutation of either of these residues. However, although used within the same studies as Cmp 1, the potential basis of binding of the antagonist CATPB was not modeled at that stage. At least in part, this reflected a lack of availability of a radiolabeled FFA2 antagonist with which to assess ligand binding and effects of receptor mutations on ligand affinity directly. Moreover, at the time of these early studies, no atomic level structure of a GPCR closely related to FFA2 was available, and therefore, models were based on the β_2_-adrenoreceptor inactive state structure (49). The relatively high similarity of FFA2 to FFA1, for which a crystal structure with a bound agonist has recently become available (31), provided a much more suitable template for homology modeling. In the FFA1 structure, the hydrogen-bonding network defined by residues Glu-172 (within extracellular loop 2), Arg-258^7.35^, and Asn-244^6.55^ is intact even with the agonist TAK-875 bound to the receptor (31). Therefore, an equivalent hydrogen-bonding network is presumed to exist in hFFA2 between Glu-166 (equivalent to Glu-172 in FFA1)–Arg-255^7.35^ (equivalent to Arg-258^7.35^) and His-242^6.55^ (equivalent to Asn-244^6.55^). In the homology model His-242^6.55^, in concert with Glu-166^ECL2^, fixes and restricts Arg-255^7.35^ in the orthosteric site of hFFA2. In hFFA1, Asn-244^6.55^ is further directed by a hydrogen bond to Ser-187^5.43^. In hFFA2, Ser-187 corresponds to the poor hydrogen bond acceptor Cys-184^5.43^, and the homology model indicates that the directing role is taken over by Tyr-94^3.37^. These important observations might play a critical role with respect to selectivity and binding of agonists and antagonists in this receptor.

Docking of Cmp 1 into the hFFA2 model resulted in a representative pose suggesting possible interactions with Arg-180^7.35^ and Arg-255^7.35^ simultaneously, both of which are necessary for Cmp 1 to bind and activate hFFA2 (Fig. 9*B*) (10). Both antagonists CATPB and GLPG0974 were able to interact with both of the arginine residues in the orthosteric binding pocket of hFFA2, and despite their structural differences, representative binding modes of the two antagonists appeared relatively similar (Fig. 10*A*). These representative poses of CATPB and GLPG0974 in hFFA2 enabled ionic interactions between GLPG0974 and Arg-180^5.39^ and between CATPB and Arg-255^7.35^. GLPG0974, having a longer more flexible carboxylate chain with which it can interact electrostatically with the presumably more flexible Arg-180^5.39^, may partly explain the observed reduced binding affinity of GLPG0974 (5.5-fold) to R180A^5.39^ hFFAR2. This is contrary to CATPB (3.5-fold decrease), which, having a shorter carboxylate chain, preferentially interacts electrostatically with Arg-255^7.35^.

A further feature in the design of GPCR-targeted ligands that has been attracting considerable attention in recent times is the issue of ligand residence time (1/*k*_off_) (50). The dwell time of a non-covalently bound ligand on a receptor can influence features such as ligand clearance and metabolism and the dosing schedule and frequency of use of a medicine (50). It was thus of interest to note that although CATPB and GLPG0974 have virtually identical affinity at hFFA2, this is achieved in distinct ways. Kinetic analysis of the binding of [^3^H]GLPG0974 in the presence of differing concentrations of CATPB demonstrated this antagonist to show both rapid association and dissociation kinetics compared with GLPG0974, which was some 10-fold slower than CATPB in both of these parameters. The slower receptor binding kinetics of GLPG0974 may to some degree be explained by its somewhat larger size and higher flexibility. Although these are relatively modest variations in ligand on and off rates, the very slow off rate of the muscarinic M_3_ receptor antagonist tiotropium has resulted in this ligand being a much greater commercial success for the treatment of chronic obstructive pulmonary disease than other M_3_ receptor antagonists of equivalently high affinity (51). Interestingly, by assessing the binding kinetics of Cmp 71 compared with its methyl ester, we established that somewhat surprisingly the ionic interaction primarily contributed to the association rate of this ligand. This indicates that the electrostatic interaction between the carboxylate of Cmp 71 and the arginine residues primarily contributes to recruit the compound and that it is less important once the other receptor binding interactions have been established. This is consistent with the observations that the electrostatic interaction is dispensable for the activity of the compound series and that one orthosteric arginine can substitute for the other. It is also in line with the finding that on-rates generally are more sensitive to modulation of the long range electrostatic charges than off-rates (52).

These studies provide novel insights into the basis of agonist and antagonist binding and are likely to provide guidance for the generation of further and improved tool compounds. Given the interest in targeting this receptor in both inflammatory (19) and metabolic diseases (44, 53), they should assist in rapid translation to assessment of such opportunities.

## Author Contributions

G. M., T. U., and B. D. H. conceived and coordinated the study. G. M., with the assistance of all others, wrote the paper; E. S. designed, performed, and analyzed the experiments shown in Figs. 2–7 and Tables 1–3 and 5; A. E. M. and B. D. H. designed, performed, and analyzed the experiments shown in Fig. 8 and Table 4. A. H. H. developed the modeling studies and the experiments of Figs. 9–11. S. K. P. synthesized novel key ligands. All authors reviewed the results and approved the final version of the manuscript.

## Footnotes

4 The abbreviations used are:

SCFA: short chain fatty acid
BRET: bioluminescence resonance energy transfer
CATPB: (*S*)-3-(2-(3-chlorophenyl)acetamido)-4-(4-(trifluoromethyl)phenyl) butanoic acid
Cmp 1: (*R*)-3-benzyl-4-(cyclopropyl-(4-(2,5-dichlorophenyl)thiazol-2-yl)amino)-4-oxobutanoic acid
eYFP: enhanced yellow fluorescent protein
FFA2: free fatty acid receptor 2
FFA3: free fatty acid receptor 3
GLPG0974: 4-[[1-(benzo[*b*]thiophene-3-carbonyl)-2-methyl-azetidine-2-carbonyl]-(3-chloro-benzyl)-amino]-butyric acid
GPCR: G protein-coupled receptor
TMD: transmembrane domain
GTPγS: guanosine 5′-3-*O*-(thio)triphosphate
h: human
m: mouse
Cmp 71: 4-(1-(benzo[*b*]thiophene-3-carbonyl)-2-methyl-*N*-(4-trifluoromethylbenzyl)azetidine-2-carboxamido)butanoic acid
MeCmp 71: methyl 4-(1-(benzo[*b*]thiophene-3-carbonyl)-2-methyl-*N*-(4-trifluoromethylbenzyl)azetidine-2-carboxamido)butanoate
Cmp 42: 4-(1-(2-(benzo[*b*]thiophen-3-yl)acetyl)-*N*-(4-chlorobenzyl)-2-methylazetidine-2-carboxamido)butanoic acid)
MeCmp 42: methyl 4-(1-(2-(benzo[*b*]thiophen-3-yl)acetyl)-*N*-(4-chlorobenzyl)-2-methylazetidine-2-carboxamido)butanoate.
